# Differences in labour market marginalisation between refugees, non-refugee immigrants and Swedish-born youth: Role of age at arrival and residency duration

**DOI:** 10.1177/14034948221079060

**Published:** 2022-03-27

**Authors:** Gerdur Geirsdottir, Ellenor Mittendorfer-Rutz, Emma Björkenstam, Lingjing Chen, Thomas E. Dorner, Ridwanul Amin

**Affiliations:** 1 Division of Insurance Medicine, Department of Clinical Neuroscience, Karolinska Institutet, Sweden; 2 Karl-Landsteiner Institute for Health Promotion Research, Austria

**Keywords:** Refugee, non-refugee immigrant, labour market marginalisation, unemployment, disability pension

## Abstract

**Aims:**

We investigated if the risk of long-term unemployment (LTU) and disability pension (DP) differs between young refugees and non-refuge immigrants compared to the Swedish-born. The role of age at arrival, duration of residency and morbidity in this association was also investigated.

**Methods:**

All 19- to 25-year-olds residing in Sweden on 31 December 2004 (1691 refugees who were unaccompanied by a parent at arrival, 24,697 accompanied refugees, 18,762 non-refugee immigrants and 621,455 Swedish-born individuals) were followed from 2005 to 2016 regarding LTU (>180 days annually) and DP using nationwide register data. Cox regression models were used to estimate crude and multivariate-adjusted (adjusted for several socio-demographic, labour market and health-related covariates) hazard ratios (aHRs) with 95% confidence intervals.

**Results:**

Compared to the Swedish-born, all migrant groups had around a 1.8-fold higher risk of LTU (range aHR=1.71–1.83) and around a 30% lower risk of DP (range aHR=0.66–0.76). Older age at arrival was associated with a higher risk of LTU only for non-refugee immigrants. Both older age at arrival and a shorter duration of residency were associated with a lower risk of DP for all migrant groups. Psychiatric morbidity had the strongest effect on subsequent DP, with no significant differences between migrant groups and the Swedish-born (range aHR=5.1–6.1).

**Conclusions:**

**Young immigrants had a higher risk of LTU and a lower risk of DP than their Swedish-born peers. No differences between the different immigrant groups were found. Age at arrival, psychiatric morbidity and duration of residency are strong determinants of being granted DP.**

## Introduction

Over the last decade, there has been a significant increase in migration, and about 10% of the World Health Organization European region populations are migrants [1]. Migration to Sweden has significantly increased since the beginning of the 21st century, and the proportion of foreign-born migrants (refugees and non-refugee immigrants) in the general population in Sweden has increased from 11.3% in 2000 to 19.7% in 2020 [2]. Of those who sought asylum in Sweden in 2019, around 30% were children <18 years of age [3].

Previous studies in Sweden showed that it is difficult for both refugees [4,5] and non-refugee immigrants [4], particularly young immigrants, to enter the labour market. Labour market marginalisation can be conceptualised in different ways. In addition to unemployment, measures of work disability (e.g. disability pension (DP)) can contribute important knowledge regarding immigrants’ marginalisation in the labour market [6]. Immigrants have been shown to have a higher risk of being granted DP, both in Sweden and in Norway [7,8]. A Swedish study showed that the risk of DP was higher for refugees than for non-refugee immigrants [4]. However, there is a knowledge gap regarding this association in younger cohorts of refugees and non-refugee immigrants. This is of interest because young immigrants might have particular difficulties entering the labour market, as establishment in the labour market is in general a crucial and difficult transition period for young individuals [6].

Immigrants also form a heterogeneous group. Refugees might differ from non-refugee immigrants regarding their subsequent risk of labour market marginalisation, as they might have experienced traumatic and stressful life events to a greater extent than non-refugee immigrants [4]. In turn, this vulnerability might increase their risk of mental ill-health. Refugees might therefore not be subject to the same positive health selection, known as the ‘healthy migrant effect’, as non-refugee migrants are [9] and consequently have lower chances of labour market establishment. Following this line of thought, it might be anticipated that refugees have a higher risk of labour market marginalisation than non-refugee immigrants. On the other hand, group-level refugees and non-refugee immigrants, particularly those who arrived as adolescents, share several characteristics that lower their chances of labour market participation compared to their host-country peers, such as lower educational level, language difficulties, less knowledge of the health care and social insurance regulations in the host country [4]. Therefore, it is also possible that labour market marginalisation may not differ between refugees and non-refugee immigrants, as these socio-demographic characteristics can be more important in determining changes for participation than potential differences in health.

Moreover, young refugees differ regarding pre- and post-migration factors. For example, unaccompanied refugee minors are expected to be more vulnerable compared to accompanied refugee minors, as they are likely to have experienced severe trauma without the possibility of support from a parent or legal guardian [10]. Consequently, unaccompanied refugees have an elevated risk for severe stress reactions and adjustment disorders compared to accompanied refugee minors [11]. It might therefore be anticipated that unaccompanied refugee minors have a higher risk of labour market marginalisation than accompanied ones. However, to the best of our knowledge, the risk of long-term unemployment (LTU) and DP in unaccompanied versus accompanied refugees has not been investigated to date.

Social integration and labour market participation also seem to change over time, improving with increasing duration of residency in the host country [12,13]. Similarly, age at arrival and the timing of entering schooling in the new host country are associated with socio-economic changes in the transition to adulthood, and this can have a long-term effect on both academic and occupational achievements [13]. This process of upward mobility can best be described by theoretical concepts of assimilation, meaning the immigrants’ adaptation to the new host society and culture [14]. Therefore, it is important to study differences in future labour market marginalisation in young immigrants based on differences regarding duration of residency and age at arrival. Moreover, psychiatric and somatic morbidity has been shown to have a differential impact on the subsequent risk of labour market marginalisation, depending on refugee and migration status [6,15,16]. Still, such analyses have not focussed on young individuals so far and have not investigated different groups of migrants and refugees, including unaccompanied and accompanied refugees. In these analyses, it is essential to consider certain confounding factors such as socio-demographic characteristics [17,18].

### Aims

We aimed to examine the risk of LTU and DP in young refugees who arrived in Sweden as unaccompanied or accompanied minors and non-refugee immigrant youth compared to their Swedish-born peers. Moreover, we examined whether age at arrival, duration of residency and specific psychiatric and somatic morbidity modify the association between migrant status and labour market outcomes.

## Methods

### Study population

The study population was defined as all individuals aged 19–25 years, residing in Sweden on 31 December 2004 (i.e. born between 1979 and 1985; *N*=737,606). Only those born in Sweden (comparison group) or those migrants (exposure groups) with complete information on their reason for settlement in Sweden were included (*n*=713,557). Those with missing information on year of immigration (*n*=146) were excluded. Immigrants who arrived in Sweden when they were between 19 and 25 years of age were also excluded (*n*=28,186). Last, of the non-refugee immigrants, we only included those who came from the same countries as the refugees (i.e. further excluding 2867 individuals). Moreover, individuals with ongoing DP in 2004 were excluded (*n*=12,560). Furthermore, individuals not residing in Sweden between 2000 and 2003 were excluded due to lack of data on health-related covariates (*n*=3193). Applying these exclusion criteria, the study population comprised 666,605 individuals, of whom 26,388 were refugees and 18,762 were non-refugee immigrants.

### Data sources

We used the unique (de-identified) Swedish personal identity number to link information from several population-based registers. The Longitudinal Integrated Database for Health Insurance and Labour Market Studies (LISA) contains data from the labour market and the educational and social sectors [19]. The Longitudinal Database for Integration Studies holds migration-related information. The Multi-Generation Register contains links to biological parents. The National Patient Register (NPR) includes information on diagnoses from inpatient and specialised outpatient health care [20], coded according to the International Classification of Diseases version 10 (ICD-10). The Cause of Death Register comprises information on all deaths. Finally, the microdata for analysis of social security provided information on the start date of DP.

### Exposure

In this study, a refugee was defined as an individual receiving a residence permit in Sweden as a refugee (according to the Geneva Convention of Refugees) or an individual who had been granted residence permit due to being ‘in need of protection’ or on ‘humanitarian grounds’ [21]. Refugees were further categorised as accompanied minors (⩽18 years) if they obtained residency because they were related to a parent who was a refugee, according to the register, or had at least one parent in the Multi-Generation Register who had received residency in Sweden the same year or one year before the refugee minor [11]. Young refugees who did not fulfil either of these two criteria but came to Sweden as minors were categorised as unaccompanied. In our cohort, 2133 and 25,225 refugees came as unaccompanied and accompanied minors, respectively. Migrants (i.e. with a birth country outside Sweden) who were not refugees were categorised as non-refugee immigrants. The comparison group included people born in Sweden.

### Outcome measures

The cohort was followed from 2005 to 2016 regarding LTU, defined as >180 annual days of unemployment according to the Swedish Labour Force Surveys [22] and newly granted DP (regardless of grade).

### The Swedish social insurance system

Permanent DP is granted to individuals 30–64 years old who suffer from long-term work disability, and the compensation can be paid in full or at 75%, 50% or 25% [19]. Individuals 19–29 years old can be granted temporary DP if their work incapacity due to injury or disease is reduced for a minimum of one year but is not considered permanent, or if compulsory education is not completed before 19 years of age [19]. The Swedish Public Employment Service registers the number of annual days with unemployment for unemployed individuals who are active job seekers and are ready to enter the labour market instantly [19]. This register includes individuals both with and without unemployment benefits. Whether an unemployed individual will get benefit, and how much, depends on other prerequisites such as a minimum of 12 months’ membership with an unemployment fund, past income, past work duration and so on. [23].

### Covariates

The following covariates were considered: (a) socio-demographic factors (measured in 2004): sex, age (years), educational level, type of living area and family situation, duration of residency and age at arrival (for migrant groups only); (b) labour market marginalisation factors (measured in 2004): history of unemployment (0, 1–180, >180 annual days), sickness absence (0, 1–90, >90 net days) and employment status (employed, unemployed with control information from employer or self-employment, unemployed without control information from employer or self-employment); (c) health-related factors (measured in 2000–2004): psychiatric morbidity at baseline was identified as a record of a main or side diagnosis from inpatient or specialised outpatient health care due to depressive disorders, anxiety disorder and other psychiatric disorders (ICD-10 codes F30–F39, F40–F49 and all other F codes except F30–F49, respectively). Somatic morbidity was measured as a record of a main or side diagnosis from inpatient or specialised outpatient health care due to musculoskeletal disorders, injury, poisoning and certain other consequences of external causes, and other somatic disorders (ICD-10 codes M00–M99, S00–T88 and all other ICD-10 codes except F, M, S and T codes, respectively). Information on socio-demographic and labour market marginalisation factors were obtained from the LISA register, and health-related factors were obtained from the NPR. Missing values in any covariate were treated as a separate category in the adjusted analyses. Table I shows the categorisation of socio-demographic, labour market marginalisation and health-related factors.

**Table I.** Socio-demographic, labour market marginalisation and health-related characteristics of the study cohort of individuals aged 19–25 years old (*N*=666605 individuals), with Swedish-born, refugees and non-refugee immigrants residing in Sweden on 31 December 2004.

	Swedish-born, *n* (%)	Refugees, *n* (%)		Non-refugee immigrants, *n* (%)	*p*-Value of chi-square test for homogeneity
		Unaccompanied refugees	Accompanied refugees		
All (row percentage)	621,455 (93.2)	1691 (0.3)	24697 (3.7)	18762 (2.8)	
*Socio-demographic factors (2004)*					
Sex					<0.001
Men	319,573 (51.4)	1010 (59.7)	12,943 (52.4)	9624 (51.3)	
Women	301,882 (48.6)	681 (40.3)	11,754 (47.6)	9138 (48.7)	
Age (years), *M* (*SD*)	22 (2.0)	22.4 (1.88)	21.7 (1.94)	21.6 (1.94)	
Education (years)					<0.001
Compulsory school (<10)	80,817 (13.0)	603 (35.7)	5903 (23.9)	5969 (31.8)	
High school (10–12)	381,333 (61.4)	748 (44.2)	13,643 (55.2)	8725 (46.5)	
College or university (>12)	155,789 (25.1)	215 (12.7)	4663 (18.9)	2564 (13.7)	
Missing	3516 (0.6)	125 (7.4)	488 (2.0)	1504 (8.0)	
Family situation					<0.001
Married/living with partner with children living at home	28,284 (4.6)	177 (10.5)	1953 (7.9)	1513 (8.1)	
Married/living with partner without children living at home	6312 (1.0)	93 (5.5)	1340 (5.4)	809 (4.3)	
Single/divorced/separated/widowed with children living at home	8036 (1.3)	60 (3.5)	465 (1.9)	452 (2.4)	
Single/divorced/separated/widowed without children living at home	443,814 (71.4)	1239 (73.3)	14,671 (59.4)	11,411 (60.8)	
Youth (⩽20 years old)	135,009 (21.7)	122 (7.2)	6265 (25.4)	4574 (24.4)	
Missing	0	0	3 (0.0)	3 (0.0)	
Type of living area^a^					<0.001
Big cities	218,159 (35.1)	937 (55.4)	11,259 (45.6)	10,824 (57.7)	
Medium-sized cities	242,304 (39.0)	589 (34.8)	9908 (40.1)	5730 (30.5)	
Small cities/villages	160,992 (25.9)	165 (9.8)	3530 (14.3)	2208 (11.8)	
*Labour market factors (2004)*					
No unemployment	445,077 (71.6)	977 (57.8)	15,191 (61.5)	12,201 (65.0)	<0.001
Unemployment 1–180 days	162,954 (26.2)	626 (37.0)	8474 (34.3)	5943 (31.7)	
Unemployment >180 days	13,424 (2.2)	88 (5.2)	1032 (4.2)	618 (3.3)	
No sickness absence	589,250 (94.8)	1578 (93.3)	23,406 (94.8)	18,058 (96.2)	<0.001
Sickness absence 1–90 net days	23,843 (3.8)	81 (4.8)	969 (3.9)	502 (2.7)	
Sickness absence >90 net days	8362 (1.3)	32 (1.9)	322 (1.3)	202 (1.1)	
Employed	371,846 (59.8)	723(42.8)	10,744 (43.5)	7186 (38.3)	<0.001
Unemployed with control information from employer or self-employment	175,050 (28.3)	414 (24.5)	7562 (30.6)	5482 (29.2)	
Unemployed without control information from employer or self-employment	(12.0)	554 (32.8)	6391 (25.9)	6094 (32.5)	
*Health-related factors (2000–2004)*					
Psychiatric morbidity^b^					<0.001
Depressive disorders	9140 (1.5)	40 (2.4)	237 (1.0)	256 (1.4)	
Anxiety disorders	8303 (1.3)	36 (2.1)	412 (1.7)	351 (1.9)	
Other psychiatric disorders	12,680 (2.0)	41 (2.4)	469 (1.9)	465 (2.5)	
Somatic morbidity^c^					0.0132
Musculoskeletal disorders	41,547 (6.7)	122 (7.2)	1626 (6.6)	1221 (6.5)	
Injury, poisoning and certain other consequences of external causes	109,888 (17.7)	323 (19.1)	4576 (18.5)	3287 (17.5)	
Other somatic disorders	195,447 (31.4)	600 (35.5)	8280 (33.5)	6464 (34.5)	
*Migration-related factors (2004)* ^d^					
Region/country of birth					<0.001
Afghanistan		65 (3.8)	112 (0.5)	474 (2.5)	
Asia (except Afghanistan, Iraq, Iran and Syria)		118 (7.0)	2970 (12.0)	4247 (22.6)	
Former Yugoslavia		286 (16.9)	10,828 (43.8)	1039 (5.5)	
Horn of Africa (Somalia, Eritrea and Ethiopia)		390 (23.1)	863 (3.5)	1772 (9.4)	
Iran		198 (11.7)	3573 (14.5)	1874 (10.0)	
Iraq		337 (19.9)	1934 (7.8)	2556 (13.6)	
Syria		82 (4.8)	938 (3.8)	306 (1.6)	
Others		215 (12.7)	3479 (14.1)	6494 (34.6)	
Duration of residency in Sweden					<0.001
0–4 years		198 (11.7)	435 (1.8)	1146 (6.1)	
5–10 years		776 (45.9)	8702 (35.2)	8140 (43.4)	
>10 years		717 (42.4)	15,560 (63.0)	9476 (50.5)	
Age at arrival (years)					<0.001
0–6		118 (7.0)	4825 (19.5)	3286 (17.5)	
7–13		761 (45.0)	15,951 (64.6)	8917 (47.5)	
14–16		418 (24.7)	3296 (13.3)	4295 (22.9)	
17–18		394 (23.3)	625 (2.5)	2264 (12.1)	
*Outcome 2005–2016*					
Disability pension	12,763 (2.1)	56 (3.3)	474 (1.9)	481 (2.6)	<0.001
Long term unemployment	92,967 (15)	660 (39)	8188 (33.2)	6081 (32.4)	<0.001

a Type of residential area: big cities – Stockholm, Gothenburg and Malmö; medium-sized cities – cities with more than 90,000 inhabitants within 30 km from the centre of the city; small cities/villages (not big or medium-sized cities).

b Defined by ICD-10 codes: depressive disorders (F30–F39), anxiety disorders (F40–F49), other psychiatric disorders (all other F codes).

c Defined by ICD-10 codes: musculoskeletal disorders (M00–99), injury, poisoning and certain other consequences of external causes (S00–T88), other somatic disorders (all other ICD-10 codes, excluding F codes).

d Not applicable for the Swedish-born.

### Statistical analyses

Descriptive statistics with frequencies and percentages were calculated for the Swedish-born, non-refugee immigrants and accompanied and unaccompanied refugees. Chi-square tests for homogeneity were conducted to test for homogeneous distributions for migrant status across the covariate categories. Crude and multivariate analyses were performed using Cox regression models of time to LTU/DP during follow-up, with calendar days used as the underlying time scale. The entry date was defined as 1 January 2005. Censoring was due to date of outcome, date of emigration, death or the end of follow-up (31 December 2016), whichever occurred first. The proportional hazard assumption was verified graphically using log-minus-log plot (plotting the log-log transformation of the survival function against survival time) over the different categories for each of the covariates and exposure variables. We assessed person-years at risk by totalling the years that the individuals lived in Sweden during the follow-up period. In the analyses of LTU as the outcome, censoring was also due to DP. First, risk estimates for unaccompanied and accompanied refugees and non-refugee immigrants were compared to the Swedish-born concerning subsequent LTU/DP. Then, the associations of the duration of residency, age at arrival, psychiatric and somatic morbidity and subsequent LTU/DP with migrant status were assessed by categorising duration of residency (<5, 5–10 and >10 years), age at arrival (0–6, 7–13, 14–16 and 17–18 years) and specific psychiatric and somatic morbidity. These categories were analogous with categories used in previous research (duration of residency and age at arrival) and different ages for school transition in Sweden (age at arrival) [24]. We examined the associations in one crude and one adjusted regression model, adjusting for age, sex, educational level, family situation, type of residential area, history of unemployment, sickness absence, employment status and specific psychiatric and somatic morbidity at baseline. The values of the covariates were taken at the cohort entry and treated as time-invariant constants in the analyses. Results are presented as crude and adjusted hazard ratios (HRs and aHRs, respectively) with 95% confidence intervals (CIs). Statistical analyses were conducted using R v4.0.4 (The R Foundation for Statistical Computing, Vienna, Austria).

### Ethics

Ethical approval was obtained from the Regional Ethical Review Board, Karolinska Institutet, Stockholm (Dnr: 2007/1762-31).

## Results

### Baseline socio-demographic, labour market and health-related characteristics

There were fairly equal proportions of women and men in all the groups (Table I), except in unaccompanied refugees, where there was a lower proportion of women (40.3%). All migrant groups had a lower educational level than their Swedish-born peers (Table I). Proportions of married/cohabiting individuals were higher among migrant groups than among the Swedish-born (Table I). A higher proportion of non-refugee immigrants lived in big cities (57.5%) compared to their Swedish-born counterparts (35.1%). All migrant groups had a higher proportion of LTU at baseline compared to the Swedish-born (Table I). Both specific psychiatric and somatic morbidity at baseline was slightly higher among unaccompanied refugees than all the other groups (Table I). The largest proportions of all migrant groups had been resident in Sweden for more than four years (98.2% of accompanied, 88.3% of unaccompanied and 93.9% of non-refugee immigrants). The most common age group arriving in Sweden was 7–13 years for all migrant groups (64.6%, 45% and 47.5% for accompanied refugees, unaccompanied refugees and non-refugee immigrants, respectively). Differences between Swedish-born individuals and the immigrant groups were statistically significant (*p*<0.001) across all covariates.

### Risk of long-term unemployment and disability pension

In the crude models, the risk of LTU for unaccompanied refugees, accompanied refugees and non-refugee immigrants was almost 2.5- to 3-fold higher compared to the Swedish-born (Table II). Adjustment for the various covariates in the adjusted models lowered the estimates to some extent (Table II). Compared to Swedish-born individuals, HRs for DP were increased for unaccompanied refugees and non-refugee migrants but similar for accompanied refugees in the crude analyses. In the multivariate-adjusted models, aHR for the migrant groups ranged from 0.66 to 0.76 (Table II).

**Table II.** Risk of long-term unemployment and disability pension in refugees and non-refugee immigrants aged 19–25 years old residing in Sweden in 2004 compared to Swedish-born individuals.

	*n* (rate per 100,000 person-years)	Crude HR (CIs)	Adjusted^a^ HR (CIs)
**Long-term unemployment**			
Swedish-born individuals	92,967 (1438)	*1 (ref.)*	*1 (ref.)*
Non-refugee immigrants	6081 (3736)	**2.45 (2.39–2.52)**	**1.71 (1.66–1.76)**
Refugees			
Unaccompanied	660 (4942)	**3.16 (2.92–3.41)**	**1.79 (1.66–1.93)**
Accompanied	8188 (3710)	**2.46 (2.40–2.51)**	**1.83 (1.79–1.88)**
**Disability pension**			
Swedish-born individuals	12,763 (178)	*1 (ref.)*	*1 (ref.)*
Non-refugee immigrants	481 (233)	**1.29 (1.17–1.41)**	**0.66 (0.60–0.72)**
Refugees			
Unaccompanied	56 (304)	**1.67 (1.29–2.18)**	**0.76 (0.59–0.99)**
Accompanied	474 (168)	0.94 (0.86–1.03)	**0.66 (0.60–0.73)**

HRs with 95% CIs in bold indicate a statistically significant association (p<0.05).

a Adjusted for age, sex, education, family situation, type of living area, history of unemployment, employment status, sickness absence, and psychiatric and somatic morbidity at baseline.

HR: hazard ratio; CI: confidence interval.

### Long-term unemployment and disability pension by migrant status and age at arrival

Table III presents the risk estimates for LTU and DP for the immigrant groups by age at arrival. The risk for LTU increased with age at arrival for non-refugee immigrants and was highest for those who had arrived in Sweden between 17 and 18 years of age (aHR=1.87, 95% CI 1.75–2.00) compared to the Swedish-born. For refugees, the risk estimates in the multivariate models did not differ across groups for age at arrival and were around 1.7- to 1.9-fold higher (Table III). On the contrary, the risk for DP differed strongly by age at arrival for both migrant groups, particularly for non-refugee immigrants, ranging from 0.41 to 1.19 for those arriving between 17 and 18 years of age and before seven years of age, respectively.

**Table III.** Risk of long-term unemployment and disability pension by migrant status and age at arrival. Individuals aged 19–25 years old residing in Sweden in 2004.

	*n* (rate per 100,000 person-years)	Crude HR (CIs)	Adjusted^a^ HR (CIs)
**Long-term unemployment**			
Age at arrival (years)			
Swedish-born individuals	92,967 (1438)	1 (ref.)	1 (ref.)
Non-refugee immigrants			
0–6	807 (2590)	**1.74 (1.63–1.87)**	**1.48 (1.38–1.59)**
7–13	2823 (3616)	**2.38 (2.29–2.47)**	**1.73 (1.66–1.80)**
14–16	1526 (4255)	**2.76 (2.62–2.90)**	**1.73 (1.64–1.82)**
17–18	925 (5230)	**3.32 (3.11–3.54)**	**1.87 (1.75–2.00)**
Refugees			
0–6	1438 (3117)	**2.08 (1.98–2.19)**	**1.73 (1.64–1.82)**
7–13	5551 (3732)	**2.47 (2.40–2.54)**	**1.84 (1.79–1.89)**
14–16	1438 (4644)	**3.03 (2.87–3.19)**	**1.94 (1.84–2.05)**
17–18	421 (5120)	**3.28 (2.98–3.61)**	**1.74 (1.58–1.92)**
**Disability pension**			
Age at arrival (years)			
Swedish-born individuals	12,763 (178)	1 (ref.)	1 (ref.)
Non-refugee immigrants			
0–6	124 (339)	**1.88 (1.57–2.24)**	**1.19 (1.00–1.42)**
7–13	208 (212)	**1.17 (1.02–1.34)**	**0.69 (0.60–0.79)**
14–16	101 (214)	1.18 (0.97–1.44)	**0.48 (0.39–0.58)**
17–18	48 (195)	1.07 (0.81–1.42)	**0.41 (0.30–0.54)**
Refugees			
0–6	101 (181)	1.01 (0.83–1.23)	**0.73 (0.60–0.89)**
7–13	313 (164)	0.92 (0.82–1.03)	**0.66 (0.59–0.74)**
14–16	89 (209)	1.17 (0.95–1.44)	**0.67 (0.55–0.83)**
17–18	27 (237)	1.31 (0.90–1.92)	**0.55 (0.38–0.80)**

HRs with 95% CIs in bold indicate statistically significant association (*p*<0.05).

a Adjusted for age, sex, education, family situation, type of living area, history of unemployment, employment status, sickness absence, and psychiatric and somatic morbidity at baseline.

HR: hazard ratio; CI: confidence interval.

### Long-term unemployment and disability pension by migrant status and duration of residency

The risk of LTU did not differ by duration of residency for the two migrant groups. Compared to Swedish-born individuals, all categories of duration of residency among both refugees and non-refugee immigrants had an almost 1.7 to 2 times higher risk for LTU (Table IV). On the other hand, the risk of DP increased considerably with increasing duration of residency in both migrant groups, particularly in non-refugee immigrants (Table IV).

**Table IV.** Risk of long-term unemployment and disability pension by migrant status and duration of residency. Individuals aged 19–25 years old residing in Sweden in 2004.

	*n* (rate per 100,000 person-years)	Crude HR (CIs)	Adjusted^a^ HR (CIs)
**Long-term unemployment**			
Duration of residency in Sweden			
Swedish-born individuals	92,967 (1438)	1 (ref.)	1 (ref.)
Non-refugee immigrants			
<5 years	414 (4191)	2.71 (2.46–2.99)	1.75 (1.58–1.93)
5–10 years	2862 (4182)	2.72 (2.62–2.82)	1.74 (1.67–1.81)
>10 years	2805 (3322)	2.20 (2.12–2.28)	1.68 (1.61–1.74)
Refugees			
<5 years	293 (5735)	3.64 (3.24–4.08)	2.04 (1.82–2.29)
5–10 years	3484 (4252)	2.79 (2.70–2.89)	1.92 (1.85–1.98)
>10 years	5071 (3449)	2.29 (2.23–2.36)	1.77 (1.72–1.82)
**Disability pension**			
Duration of residency in Sweden			
Swedish-born individuals	12,763 (178)	1 (ref.)	1 (ref.)
Non-refugee immigrants			
<5 years	25 (199)	1.10 (0.74–1.62)	**0.29 (0.20–0.44)**
5–10 years	194 (217)	**1.20 (1.04–1.38)**	**0.54 (0.46–0.62)**
>10 years	262 (250)	**1.38 (1.22–1.56)**	0.89 (0.79–1.01)
Refugees			
<5 years	18 (253)	1.41 (0.89–2.23)	**0.38 (0.24–0.60)**
5–10 years	198 (182)	1.02 (0.89–1.17)	**0.64 (0.56–0.74)**
>10 years	314 (170)	0.95 (0.85–1.06)	**0.72 (0.64–0.81)**

HRs with 95% CIs in bold indicate statistically significant association (p<0.05).

a Adjusted for age, sex, education, family situation, type of living area, history of unemployment, employment status, sickness absence, and psychiatric and somatic morbidity at baseline.

HR: hazard ratio; CI: confidence interval.

The associations between psychiatric and somatic morbidity with subsequent LTU and DP are shown in Supplemental Tables 2 and 3. While there was a slightly higher risk of LTU for Swedish-born individuals with psychiatric or somatic morbidity compared to those without, such morbidity did not seem to alter the risk of LTU for either migrant group. On the contrary, somatic and particularly psychiatric morbidity increased the risk of DP for both the Swedish-born and the migrant groups, leading to HRs of 5.1–6.1 for those with psychiatric morbidity at baseline (compared to Swedish-born individuals without psychiatric morbidity).

### Sensitivity analyses

We also conducted a sensitivity analysis, excluding individuals who were granted residence permits due to ‘in need of protection’ and on ‘humanitarian grounds’ from the refugee category (63 unaccompanied refugees and 1580 accompanied refugees were included). This analysis showed a similarly higher risk estimates of LTU in the refugee groups compared to our main analysis (Supplemental Table 1). However, the analysis on DP as outcome lacked statistical power to show comparable results. In all analytical models for LTU, we ran separate competing risk models, with DP as the competing event. These sensitivity analyses showed nearly identical risk estimates to our main analyses (data not shown).

## Discussion

### Main findings

Young refugees (unaccompanied and accompanied) and non-refugee immigrants had around a 1.8-fold higher risk of LTU and approximately 30% lower risk of DP compared to Swedish-born peers, meaning that no differences between the three migrant groups emerged in terms of this relation. Age at arrival and duration of residency did not seem to affect the risk of LTU in the migrant groups, with the exception of an increase in risk with increasing age at arrival for non-refugee immigrants. On the contrary, the risk of DP decreased considerably with older age at arrival and a shorter duration of residency and strongly increased in the case of psychiatric morbidity at baseline for both migrant groups.

### Long-term unemployment and disability pension in young migrants

This study showed a higher risk for LTU, for both refugees and non-refugee immigrants with fairly similar risk estimates compared to the Swedish-born. Previous studies considering all age groups with a shorter follow-up period (four years) have reported 1.8–2.2 times and 2.4 times higher risk of LTU for refugees [4,16] and non-refugee immigrants [4], respectively, compared with the host population, showing a slightly higher risk for non-refugee immigrants than refugees. However, our study showed that these discrepancies exist in almost the same magnitude for a younger cohort of refugees and non-refugee immigrants. Migrant groups are generally more likely to be marginalised in the labour market compared to individuals in the host population [25], which might make them more vulnerable when it comes to stable employment. Low educational level and language barriers [18] might lead to a longer time for refugees and non-refugee immigrants to establish themselves in the labour market, and with such disadvantage, it is challenging for them to reach equal labour market positions as their Swedish-born peers [26]. As estimates for subsequent labour market marginalisation were similar for refugees and non-refugee migrants, there does not seem to be any strong evidence for any potential differences in the impact of socio-economic adversities, including childhood poverty, between the two migrant groups. Contrary to the finding for long-term unemployment, we found a lower risk of another measure of labour market marginalisation – that is, of being granted DP – for both refugees and non-refugee immigrants (with fairly similar risk estimates) compared to Swedish-born individuals. Previous studies reported a higher risk of DP for refugees [4,16] but a lower risk of DP for adult non-refugee immigrants [4] and young migrants [6] compared to the Swedish-born. Our results regarding the risk of DP for non-refugee immigrants are in line with previous results [4,6]. Young migrants generally have a higher risk of mental disorders compared to the host population [27]. Still, the risk of DP was lower for all migrant groups in our study. As granting of DP often entails a long period of medical assessments, migrants might find it more difficult than their Swedish-born counterparts to fulfil the prerequisites of being granted DP because of barriers to accessing health care for migrants (e.g. language difficulties, inadequate health literacy and lack of knowledge of the social insurance system) [28]. These obstacles might be even stronger for younger than older migrants. More research on the pathways to DP in young migrants is warranted in order to prevent early exclusion from the labour market.

Interestingly, contrary to our expectation, a higher burden of mental ill-health and traumatic past in refugees compared to non-refugee immigrants [4,29] did not affect the pathways to labour market marginalisation differently in these young migrant groups. This may suggest that the factors influencing labour market marginalisation in migrant youth are quite independent of the migration-related characteristics, and therefore uniform labour market policy measures may help different migrant groups equally, irrespective of their inherent heterogeneity.

### Unaccompanied and accompanied refugees

Both unaccompanied and accompanied minors had a higher risk of LTU and a lower risk of DP compared to their Swedish-born peers. Given that unaccompanied refugees have a higher risk for morbidity (as shown in Table I) and for psychiatric disorders such as post-traumatic stress disorder, depression and anxiety compared to accompanied refugees [30], we anticipated that they would be more marginalised in the labour market than the accompanied refugees. However, our results did not support this hypothesis. This may suggest that the factors differentiating these refugee groups such as risk of mental disorder, lack of parental support and so on had a minor influence on their risk of LTU and DP. It is also likely that an easier social integration and language acquisition facilitated by their host families might compensate for the risk associated with higher morbidity levels in unaccompanied refugees in relation to the risk of subsequent labour market marginalisation.

### Age at arrival and duration of residency

Age at arrival and duration of residency did not seem to have a strong influence on the risk of unemployment, but it did affect DP. Older age at arrival and shorter duration of residency were associated with considerably lower risk estimates for DP. A previous study that did not differentiate between refugees and non-refugee immigrants reported a lower risk of DP for newly arrived immigrants aged 16–64 years, with an increasing risk of DP with longer duration of residency [7]. As we stratified for refugees and non-refugee immigrants, our study adds additional information on how age at arrival and duration of residency modify the risk of subsequent DP in a younger cohort of several migrant subgroups.

Migrating at an older age and a shorter duration of residency limits the possibility of developing one’s language skills, which in turn is related to educational and work opportunities. Similarly, inadequate language skill is one of the key barriers to accessing health care, and therefore refugees and non-refugee immigrants who came to Sweden at an older age and had a shorter duration of residency probably found it more difficult to be granted DP. Furthermore, knowledge of the Swedish social insurance system also improves with duration of residency [31]. Migrants who entered the host country at an older age and those with a shorter duration of residency need special attention concerning health and social outcomes.

### Psychiatric and somatic morbidity

We found that somatic and particularly psychiatric morbidity was associated with higher risk estimates for LTU in Swedish-born compared to Swedish-born without such morbidity, but that the presence of such morbidity did not affect the subsequent risk of LTU in any of the migrant groups. This is in line with previous reports on adult refugees and young immigrants resettled in Sweden [6,16]. One reason behind these findings might be that young refugees and non-refugee migrants have a high risk for LTU which is not exacerbated by psychiatric or somatic morbidity. On the contrary, somatic and particularly mental disorders were associated with considerably higher estimates for DP in the Swedish-born as well as both migrant groups. While one previous study on young migrants found similar results – namely, no difference in the elevated estimates for Swedish-born individuals and migrants with mental disorders for subsequent DP [6] – another study found much higher estimates of subsequent DP for adult refugees with mental disorders than for the Swedish-born with similar disorders [16]. As previously discussed, obstacles in the health care and social insurance system on the pathways to DP might be stronger for younger than older migrants not only in general but also in particular for those with mental disorders.

### Methodological considerations

Strengths of this study include using Swedish nationwide registers with high completeness, which gives the possibility of including the whole population with basically no loss to follow-up [19,32]. We were able to consider several socio-demographic, labour market marginalisation and health-related factors at baseline as covariates. Limitations include that it was only possible to retrieve information on health care from specialised outpatient and inpatient health care, which mostly cover more severe cases of health problems. Data from primary care was not available. Moreover, we could not use time-varying health care–related covariates in our analysis due to lack of data. This may have led to residual confounding. Another source of residual confounding could be the considerable proportion of ‘missing information’ on educational level. We therefore grouped individuals with such missing information as a separate category, as in previous studies [6,7,11,24]. It is plausible that this group of individuals belong to the group with ‘compulsory school’ educational level (<10 years) [8]. Furthermore, we could not determine (un)accompanied refugee status if refugee children were accompanied by someone other than their parent (e.g. sibling, grandparent, etc.) due to lack of such data in the multigeneration resister. In addition, the reference group (Swedish-born individuals) in this study included second-generation migrants born in Sweden who could fare worse regarding health and economic outcomes than individuals who migrated as children (one-and-a-half-generation) [33]. However, as they comprise a small fraction of the population, it is unlikely that this small proportion has had a considerable effect on the estimates reported in this study. In 2000, Swedish-born individuals with two foreign-born parents comprised 3.2% of the population [34]. Also, LTU was measured from register data, which include only active jobseekers with or without benefits. Therefore, our results cannot be generalised to other individuals who are outside the labour market such as traditional homemakers, students, asylum seekers, welfare recipients and so on. Our results are mainly generalisable to other high-income countries with similar health and social welfare systems. Another limitation was that our measurements of LTU during the follow-up (2005–2016) could have been different before and after the global economic recession that started in 2008. The youth in Sweden were also heavily affected by the financial crisis in early 1990s. We could not disentangle if such differences have affected the results differentially for the migrant groups and the Swedish-born population in our study., Therefore, the generalisability of our results could be limited to this specific time period. Future studies on labour market marginalisation among youth should consider different time periods in their analyses.

## Conclusions

Young unaccompanied and accompanied refugees and non-refugee immigrants had higher risks of LTU and lower risks of DP compared to Swedish-born peers, with similar risk estimates for both outcomes across all migrant groups. Age at arrival and duration of residency did not seem to affect the risk of LTU in refugees, but older age at arrival was associated with a higher risk of unemployment for non-refugee immigrants. On the contrary, the risk of DP decreased considerably with older age at arrival and a shorter duration of residency for both migrant groups, more prominently among non-refugee immigrants. Morbidity, particularly psychiatric morbidity, had a strong impact on the risk of being granted DP but not on the risk of LTU for migrants. The results related to a higher risk for LTU and a lower risk of DP for migrant subgroups highlight the importance of applying different measures of labour market marginalisation in studies on migrants, as well as setting clear policies on labour market-related efforts to decrease the risk of early social exclusion for migrant groups.

## Supplemental Material

sj-docx-1-sjp-10.1177_14034948221079060 – Supplemental material for Differences in labour market marginalisation between refugees, non-refugee immigrants and Swedish-born youth: Role of age at arrival and residency duration Click here for additional data file. Supplemental material, sj-docx-1-sjp-10.1177_14034948221079060 for Differences in labour market marginalisation between refugees, non-refugee immigrants and Swedish-born youth: Role of age at arrival and residency duration by Gerdur Geirsdottir, Ellenor Mittendorfer-Rutz, Emma Björkenstam, Lingjing Chen, Thomas E. Dorner and Ridwanul Amin in Scandinavian Journal of Public Health
