# Supplementary material for: Differences in labour market marginalisation between refugees, non-refugee immigrants and Swedish-born youth: Role of age at arrival and residency duration
Source: Scand J Public Health. 2022 Mar 27;51(3):391–402. doi: 10.1177/14034948221079060 (PMC10251448; doi:10.1177/14034948221079060)
Supplement: sj-docx-1-sjp-10.1177_14034948221079060 – Supplemental material for Differences in labour market marginalisation between refugees, non-refugee immigrants and Swedish-born youth: Role of age at arrival and residency duration [file sj-docx-1-sjp-10.1177_14034948221079060.docx]

**Supplementary table S1** Risk of long-term unemployment and disability pension in refugees, aged 19-25 years old residing in Sweden in 2004, compared to Swedish-born individuals. Hazard ratios (HRs) with 95% confidence intervals (CIs).

| **Long-term unemployment** | **Crude**  **HR (CIs)** | **Adjusted^a^ HR (CIs)** |
| --- | --- | --- |
|  |  |  |
| Swedish-born individuals | 1 (REF) | 1 (REF) |
| **Refugees^b^** |  |  |
| Unaccompanied | **3.26 (2.24 – 4.76)** | **2.19 (1.50 – 3.20)** |
| Accompanied | **2.14 (1.96 – 2.34)** | **1.67 (1.53 – 1.83)** |
|  |  |  |
| **Disability pension** |  |  |
| Swedish-born individuals | 1 (REF) | 1 (REF) |
| **Refugees^b^** |  |  |
| Unaccompanied^c^ | ^-^ | ^-^ |
| Accompanied | 1.06 (0.76 - 1.49) | 0.77 (0.55 - 1.08) |

HRs with 95% CIs in bold indicate statistically significant association (p-value <0.05)

^a^ Adjusted for age, sex, education, family situation, type of living area, history of unemployment, employment status, sickness absence, and psychiatric and somatic morbidity at baseline

^b^ Refugees who were granted residence permit due to “in need of protection” and on “humanitarian grounds” were excluded (n=63 unaccompanied and 1580 accompanied refugees were included)

^c^ No unaccompanied refugees aged 19-25 years were granted disability pension during the follow-up in 2005-2016

**Supplementary table S2**. Risk of long-term unemployment and disability pension by migrant status and psychiatric morbidity. Individuals aged 19-25 years old residing in Sweden in 2004. Hazard ratios (HRs) with 95% confidence intervals (CIs).

|  | **N (rate per 100,00 person-years)** | **Crude** | **Adjusted** |
| --- | --- | --- | --- |
| **Long-term unemployment** |  | *HR (CIs)* | *HR (CIs)* |
| **Swedish-born individuals** |  |  |  |
| No psychiatric disorders | 85591 (1379) | 1 (REF) | 1 (REF) |
| Depressive disorders | 2087 (2772) | **1.88 (1.80 – 1.96)** | **1.18 (1.13 – 1.23)** |
| Anxiety disorders | 2059 (2877) | **1.98 (1.89 – 2.06)** | **1.24 (1.18 – 1.29)** |
| Other psychiatric disorders | 3230 (2898) | **2.00 (1.93 – 2.07)** | **1.24 (1.20 – 1.28)** |
| **Non-refugee immigrants** |  |  |  |
| No psychiatric disorders | 107 (6326) | **2.51 (2.44 – 2.57)** | **1.73 (1.68 – 1.78)** |
| Depressive disorders | 130 (4750) | **3.97 (3.28 – 4.80)** | **2.06 (1.70 – 2.49)** |
| Anxiety disorders | 174 (5111) | **3.14 (2.64 – 3.72)** | **1.85 (1.56 – 2.20)** |
| Other psychiatric disorders | 5670 (3660) | **3.30 (2.85 – 3.83)** | **1.72 (1.48 – 2.00)** |
| **Refugees** |  |  |  |
| No psychiatric disorders | 103 (5070) | **2.55 (2.49 – 2.61)** | **1.85 (1.81 – 1.89)** |
| Depressive disorders | 205 (6339) | **3.27 (2.70 – 3.97)** | **1.74 (1.43 – 2.11)** |
| Anxiety disorders | 227 (6295) | **4.08 (3.56 – 4.68)** | **2.11 (1.84 – 2.43)** |
| Other psychiatric disorders | 8313 (3692) | **4.02 (3.52 – 4.57)** | **1.92 (1.68 – 2.19)** |
|  |  |  |  |
| **Disability pension** |  |  |  |
| **Swedish-born individuals** |  |  |  |
| No psychiatric disorders | 8381 (122) | 1 (REF) | 1 (REF) |
| Depressive disorders | 1814 (2006) | **15.66 (14.89 – 16.48)** | **5.24 (4.95 – 5.53)** |
| Anxiety disorders | 1203 (1387) | **11.00 (10.36 – 11.69)** | **4.21 (3.95 – 4.48)** |
| Other psychiatric disorders | 1365 (1014) | **8.08 (7.63 – 8.56)** | **3.38 (3.18 – 3.59)** |
| **Non-refugee immigrants** |  |  |  |
| No psychiatric disorders | 53 (2169) | **1.26 (1.13 – 1.41)** | **0.57 (0.51 – 0.64)** |
| Depressive disorders | 41 (1119) | **16.70 (12.75 – 21.87)** | **4.32 (3.29 – 5.66)** |
| Anxiety disorders | 80 (1755) | **8.90 (6.55 – 12.10)** | **2.72 (2.00 – 3.70)** |
| Other psychiatric disorders | 307 (157) | **13.70 (10.99 – 17.08)** | **4.14 (3.31 – 5.16)** |
| **Refugees** |  |  |  |
| No psychiatric disorders | 52 (1900) | 1.00 (0.90 – 1.12) | **0.61 (0.55 – 0.68)** |
| Depressive disorders | 51 (1068) | **14.82 (11.28 – 19.46)** | **4.38 (3.33 – 5.75)** |
| Anxiety disorders | 72 (1375) | **8.53 (6.47 – 11.23)** | **2.55 (1.93 – 3.35)** |
| Other psychiatric disorders | 355 (123) | **10.82 (8.58 – 13.65)** | **3.97 (3.14 – 5.01)** |

HRs with 95% CIs in bold indicate statistically significant association (p-value <0.05)

^a^ Adjusted for age, sex, education, family situation, type of living area, history of unemployment, employment status, sickness absence, and somatic morbidity at baseline

**Supplementary table S3**. Risk of long-term unemployment and disability pension by migrant status and somatic morbidity. Individuals aged 19-25 years old residing in Sweden in 2004. Hazard ratios (HRs) with 95% confidence intervals (CIs).

|  | **N (rate per 100,00 person-years)** | **Crude** | **Adjusted** |
| --- | --- | --- | --- |
| **Long-term unemployment** |  | *HR (CIs)* | *HR (CIs)* |
| **Swedish-born individuals** |  |  |  |
| No somatic disorders | 38095 (1320) | *1 (REF)* | *1 (REF)* |
| Musculoskeletal disorders | 6371 (1492) | 1.12 (1.09 – 1.15) | *0.99 (0.96 – 1.01)* |
| Injury, poisoning and certain other consequences of external causes | 18375 (1635) | 1.23 (1.21 – 1.25) | *1.01 (1.00 – 1.03)* |
| Other somatic disorders | 30126 (1487) | ***1.12 (1.10 – 1.14)*** | ***1.05 (1.04 – 1.07)*** |
| **Non-refugee immigrants** |  |  |  |
| No somatic disorders | 421 (4072) | **2.29 (2.19 – 2.39)** | **1.63 (1.56 – 1.70)** |
| Musculoskeletal disorders | 1230 (4428) | **2.89 (2.62 – 3.18)** | **1.75 (1.59 – 1.93)** |
| Injury, poisoning and certain other consequences of external causes | 2237 (4000) | **3.13 (2.95 – 3.31)** | **1.80 (1.70 – 1.91)** |
| Other somatic disorders | 2193 (3191) | **2.85 (2.73 – 2.97)** | **1.84 (1.76 – 1.92)** |
| **Refugees** |  |  |  |
| No somatic disorders | 610 (4041) | **2.43 (2.35 – 2.52)** | **1.85 (1.78 – 1.92)** |
| Musculoskeletal disorders | 1875 (4538) | **2.88 (2.66 – 3.12)** | **1.83 (1.69 – 1.98)** |
| Injury, poisoning and certain other consequences of external causes | 3048 (3862) | **3.21 (3.07 – 3.36)** | **1.87 (1.79 – 1.96)** |
| Other somatic disorders | 3315 (3358) | **2.77 (2.67 – 2.88)** | **1.89 (1.82 – 1.97)** |
|  |  |  |  |
| **Disability pension** |  |  |  |
| **Swedish-born individuals** |  |  |  |
| No somatic disorders | 3559 (112) | *1 (REF)* | *1 (REF)* |
| Musculoskeletal disorders | 1574 (332) | **2.95 (2.78 – 3.13)** | **1.64 (1.55 – 1.75)** |
| Injury, poisoning and certain other consequences of external causes | 3072 (244) | **2.17 (2.06 – 2.27)** | **1.21 (1.15 – 1.27)** |
| Other somatic disorders | 4558 (202) | **1.80 (1.73 – 1.88)** | **1.35 (1.29 – 1.42)** |
| **Non-refugee immigrants** |  |  |  |
| No somatic disorders | 65 (485) | **1.33 (1.12 – 1.59)** | **0.59 (0.50 – 0.71)** |
| Musculoskeletal disorders | 129 (351) | **4.26 (3.33 – 5.44)** | **1.37 (1.07 – 1.75)** |
| Injury, poisoning and certain other consequences of external causes | 158 (219) | **3.09 (2.59 – 3.69)** | **0.98 (0.82 – 1.16)** |
| Other somatic disorders | 129 (153) | **1.93 (1.65 – 2.27)** | **0.77 (0.66 – 0.91)** |
| **Refugees** |  |  |  |
| No somatic disorders | 71 (362) | 0.92 (0.77 – 1.10) | **0.59 (0.49 – 0.70)** |
| Musculoskeletal disorders | 141 (253) | **3.20 (2.53 – 4.04)** | **1.39 (1.10 – 1.76)** |
| Injury, poisoning and certain other consequences of external causes | 190 (187) | **2.24 (1.90 – 2.65)** | **0.89 (0.75 – 1.05)** |
| Other somatic disorders | 128 (104) | **1.66 (1.44 – 1.92)** | 0.87 (0.75 – 1.01) |

HRs with 95% CIs in bold indicate statistically significant association (p-value <0.05)

^a^ Adjusted for age, sex, education, family situation, type of living area, history of unemployment, employment status, sickness absence, and psychiatric morbidity at baseline.
